# Human acellular dermal matrix versus Integra® for single-stage oncologic scalp reconstruction: A multicentre cohort study

**DOI:** 10.1016/j.jpra.2026.07.026

**Published:** 2026-07-23

**Authors:** Lorena Gallego, Laura Rúa, Cristina Sánchez, Marta Pevida, Álvaro Meana, David Robla, Felipe Salinas, Matías F. Escobedo, Luis Junquera

**Affiliations:** a Department of Oral and Maxillofacial Surgery. Hospital Universitario de Cabueñes, 33394, Gijón, Spain; b Department of Plastic and Reconstructive Surgery, Cruz Roja Hospital, 33202, Gijón, Spain; c Instituto Universitario Fernández-Vega, Fundación de Investigación Oftalmológica, University of Oviedo, Oviedo, Spain; d Instituto de Investigación Sanitaria del Principado de Asturias (ISPA), Oviedo, Spain; e Centro de Investigación Biomédica en Red en Enfermedades Raras (CIBERER), Instituto de Salud Carlos III (ISCIII), Madrid, Spain; f Tissue Engineering Unit, Centro Comunitario de Sangre y Tejidos de Asturias (CCST), Oviedo, Spain; g Department of Plastic and Reconstructive Surgery, Central University Hospital, 33011, Oviedo, Spain; h Department of Surgery and Specialties, University of Oviedo, 33006 Oviedo, Spain

**Keywords:** Scalp reconstruction, Acellular dermal matrix, Dermal substitute, Skin graft, Nonmelanoma skin cancer, Head and neck oncology, Tissue engineering, Cost-effectiveness

## Abstract

**Background:**

Reconstruction of scalp defects after oncologic resection remains challenging because of limited tissue mobility and frequent periosteal involvement. In elderly or frail patients, complex procedures such as large local flaps or free tissue transfer carry significant morbidity. Acellular dermal matrices (ADMs) offer an alternative for single-stage wound coverage. This multicentre study compared a tissue bank–manufactured human ADM with the commercial dermal regeneration template Integra®.

**Methods:**

A multicentre observational study was conducted between January 2023 and June 2025 at two university hospitals. Sixteen patients underwent scalp reconstruction after excision of nonmelanoma skin cancer: six received cadaveric human ADM and ten Integra®. The primary outcome was time to complete epithelialization. Secondary outcomes included graft integration, postoperative complications, operative time, and material cost.

**Results:**

All grafts integrated successfully, with no failures. Mean healing time was shorter with ADM (1.8 ± 0.3 months) than with Integra® (3.5 ± 1.4 months; mean difference 1.7 months, 95% CI 0.48–2.92; p = 0.01; Cohen's d = 1.54). One postoperative infection occurred in the ADM group and resolved with oral antibiotics; none occurred with Integra®. Operative times were comparable. Material cost was approximately 72% lower with tissue bank–derived ADM (€500 vs. €1,759 per reconstruction).

**Conclusions:**

In this multicentre pilot study, single-stage scalp reconstruction using tissue bank–manufactured human ADM achieved successful graft integration, faster healing, and substantially lower material cost than Integra®. Given the small sample size (n = 16) and observational design, these findings should be considered preliminary and hypothesis-generating.

## Introduction

The incidence of nonmelanoma skin cancers (NMSC) of the scalp continues to rise globally, particularly among elderly populations with chronic sun exposure. Wide local excision is the cornerstone of treatment for basal cell carcinoma (BCC) and squamous cell carcinoma (SCC), but the resulting defects frequently require complex reconstruction due to the limited elasticity of the scalp, involvement of the periosteum, and restricted availability of adjacent tissue for local recruitment.

Traditionally, scalp defects have been reconstructed using local rotation flaps, pericranial flaps, or free tissue transfer. Although these techniques provide reliable coverage, they involve extensive surgical procedures associated with longer operative times and higher morbidity, even for relatively small defects.^1^, ^2^, ^3^ This challenge is particularly relevant in elderly or medically frail patients, in whom simplified and less invasive reconstructive strategies are desirable to reduce perioperative risk.

Acellular dermal matrices (ADMs), also referred to as decellularized dermis, have emerged as promising biomaterials for reconstructive surgery. These matrices are derived from cadaveric or xenogeneic skin and processed through decellularization techniques that remove immunogenic cellular components while preserving the extracellular matrix architecture. The resulting scaffold supports neovascularization, cellular migration, and epithelialization, thereby facilitating tissue integration and wound healing.^4^ Their application in head and neck oncologic reconstruction has been described in the literature,^5^^,^^6^ yet data comparing human cadaveric ADMs with commercial xenogeneic dermal substitutes in scalp oncology remain limited.

Tissue banks have expanded their processing capabilities to include acellular dermal products, enabling the development of affordable biological scaffolds within publicly funded health systems.^7^^,^^8^

The integration of tissue-engineering principles into circular economy frameworks within hospital networks represents an emerging and cost-conscious paradigm in reconstructive surgery. A more detailed discussion of the circular-economy rationale has been relocated to the Discussion section

The aim of this study was to evaluate the clinical outcomes of a novel human ADM developed and manufactured by a regional tissue bank within the Spanish public health system, used for single-stage reconstruction of oncologic scalp defects, and to compare these outcomes with those achieved using a commercially available dermal regeneration template (Integra®).

## Materials and methods

### Preparation of the acellular dermal matrix

Skin was obtained from altruistic donors aged 14–75 years through the regional tissue bank according to institutional procurement protocols approved by the regional ethics committee. Donors with active infection, neoplastic disease, inflammatory skin conditions, dermatitis, atypical nevi, or prior radiotherapy were excluded. Full-thickness skin grafts were harvested from the anterior thigh or lumbar regions using electric dermatomes with a maximum opening of 0.03 inches. Grafts were stored in sterile saline at −80°C until processing at the Community Blood and Tissue Center of the Principality of Asturias (CBTH), a non-profit institution integrated within the Spanish National Health System.

**Figure 1:**
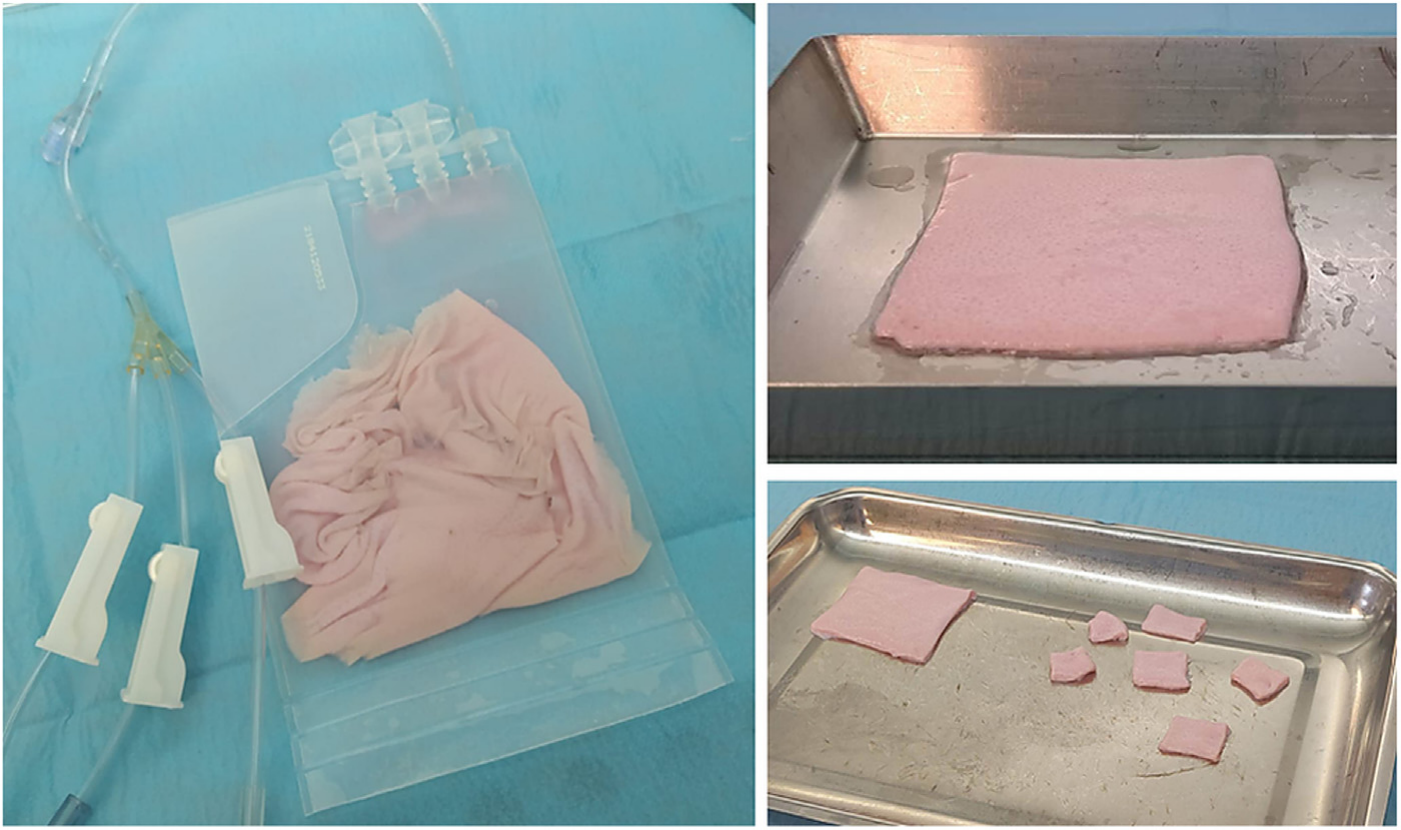
Manufacturing process and macroscopic appearance of the acellular dermal matrix produced at the Community Blood and Tissue Center of the Principality of Asturias.

All processing steps were performed under sterile conditions. The decellularization protocol comprised enzymatic digestion with Dispase II (2 mg/mL; Sigma-Aldrich) at 37°C for 24 hours, followed by three washes in distilled water and ten freeze–thaw cycles over 48 hours. Matrices were subsequently incubated in 10% NaCl at 4°C for 24 hours and subjected to two enzymatic digestions using trypsin/EDTA (0.05/0.02%; Gibco), with triple washing steps. Final ADMs were preserved by cryopreservation at −80°C or lyophilization (LyoBeta mini, Telstar-Azbil). Microbiological cultures were systematically obtained at multiple processing stages to confirm sterility. The manufacturing workflow and macroscopic appearance of the processed ADM are shown in Figure 1.

### Study design and patient selection

A multicentre observational study was conducted at two public university hospitals between January 2023 and June 2025. The study included a consecutive cohort of patients undergoing reconstruction of scalp defects following oncologic excision. Sixteen patients were included and divided into two groups: six reconstructed with the tissue bank–derived human ADM and ten with the commercially available Integra® dermal regeneration template (LifeSciences Corporation, NJ, USA).

**Figure 2:**
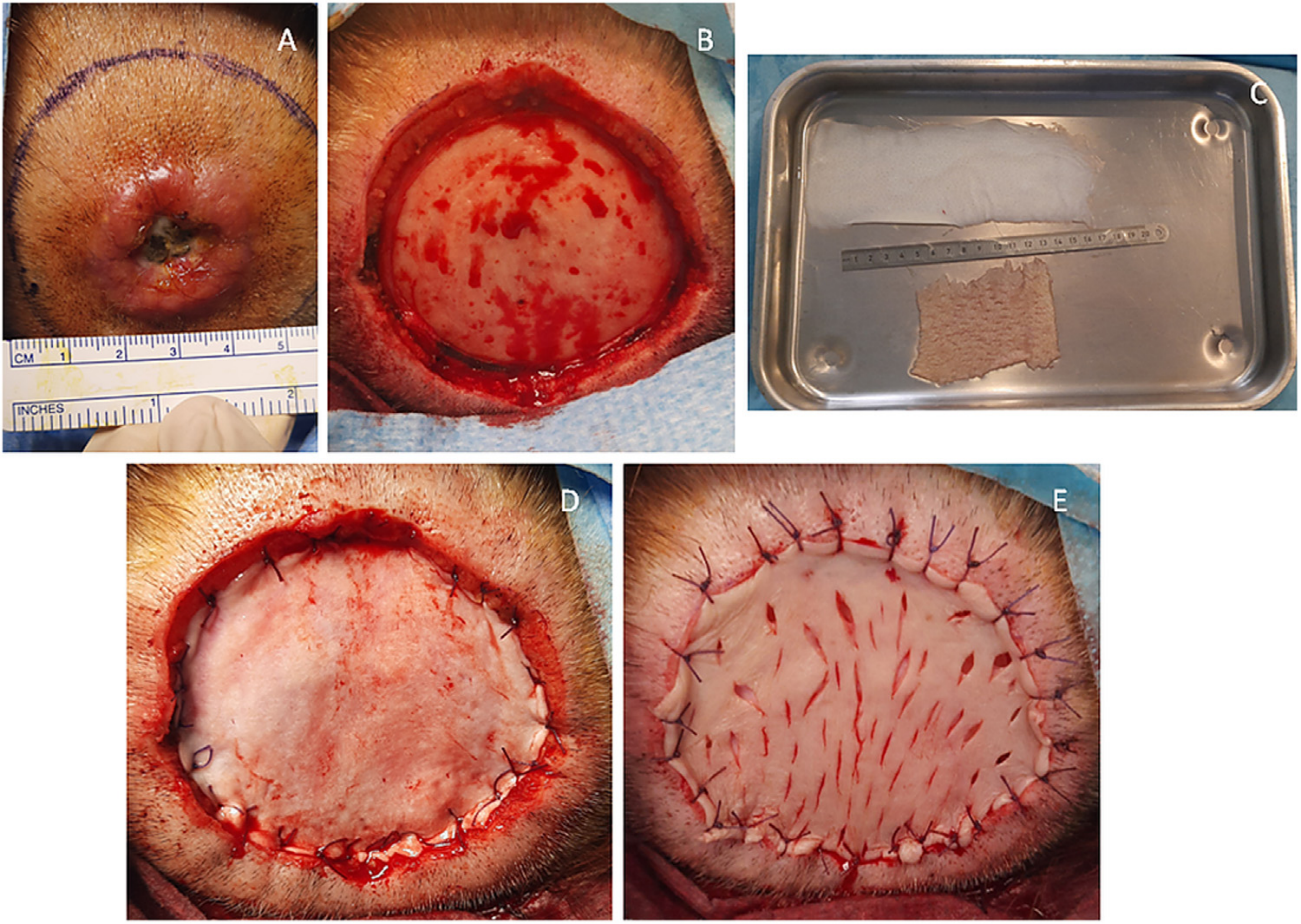
Preoperative and intraoperative photographs. (A) Preoperative scalp tumor. (B) Full-thickness excision including periosteum. (C) ADM allograft and split-thickness skin graft ready for application. (D) ADM tailored to defect dimensions and placed directly onto exposed cortical bone. (E) Meshed split-thickness skin graft applied over the ADM.

Inclusion criteria were: (1) adult patients undergoing surgery for scalp defects following oncologic excision; (2) defects not amenable to primary closure; and (3) complete postoperative follow-up until wound healing. Exclusion criteria included active surgical site infection, uncontrolled systemic diseases potentially affecting wound healing, and loss to follow-up. Treatment allocation was based on product availability and surgeon preference during the early clinical implementation phase of the tissue bank–manufactured ADM. The study was conducted in accordance with the Declaration of Helsinki and approved by a regional ethics committee (approval code: 2021.547). Written informed consent was obtained from all participants (Figure 2).

Baseline comparability between groups was assessed for age, sex, tumour diagnosis and defect size, which did not differ significantly (see Results). Additional variables that may influence wound healing and between-group comparability (smoking status, diabetes mellitus, peripheral vascular disease, anticoagulant or antiplatelet therapy, immunosuppression, previous radiotherapy, ASA physical status classification, and the Charlson Comorbidity Index) were not systematically collected in this pilot cohort. This is acknowledged as a limitation in the discussion, and their prospective collection is recommended for future confirmatory studies.

### Surgical technique

All procedures were performed by surgeons experienced in reconstructive surgery using a standardized oncologic excision protocol. Scalp lesions were excised with oncologically appropriate margins, and the periosteum was removed when oncologically required. The reconstructive approach differed according to the dermal substitute used. In the tissue bank–derived human ADM group, the ADM sheet (∼1.5 mm thick) was tailored to the defect and placed directly onto the exposed cortical bone, followed immediately by application of a meshed split-thickness skin graft (0.4 mm) harvested from the thigh, completing the reconstruction in a single operative stage. In the Integra® group, the bilayer dermal regeneration template was trimmed to the defect and applied with the collagen–glycosaminoglycan layer in contact with the wound bed and the silicone layer facing outward. After clinical evidence of neodermis formation, the silicone layer was removed and a meshed split-thickness skin graft was applied in a second stage. In both groups, the reconstructive material was secured with interrupted 3-0 Vicryl sutures, and a non-adherent greasy mesh with compressive dressing was maintained for seven days.

Defect area was estimated intraoperatively as an ellipse calculated from the two maximal perpendicular diameters, measured with a sterile ruler. Surgical margins were standardised according to tumour type and current oncologic guidelines (≥4 mm for basal cell carcinoma and ≥6 mm for squamous cell carcinoma). When the periosteum required excision because of oncologic involvement, cortical bone fenestration (multiple 2‑mm burr holes exposing the diploic space) was performed to promote granulation tissue formation prior to application of the ADM or Integra®.

### Outcomes and statistical analysis

Postoperative follow-up visits were scheduled at weeks 1, 2, 4, and 12, and at 6 months. Variables collected included demographic data, index operative time, postoperative complications (infection or graft failure), time to complete epithelialization, and material reconstruction cost per patient. Statistical analysis was performed using SPSS v28.0 (IBM Corp.). Normality of continuous variables was assessed with the Shapiro-Wilk test. Normally distributed continuous variables were compared using independent-samples Student's t-tests, and non-normally distributed continuous variables were assessed using the Mann-Whitney U test; categorical variables were compared using Fisher's exact test. Mean differences with 95% confidence intervals and Cohen's d effect size were calculated. A post-hoc power analysis was retained only as contextual information for the primary endpoint. Statistical significance was set at p < 0.05. Cost analysis included direct material costs only.

Complete epithelialization was defined as full re-epithelialization of the wound bed, without exudate, need for further dressings, or residual open areas, confirmed on clinical examination. Healing status was assessed by the treating surgical team at each scheduled visit and supported by standardised clinical photography; assessment was not performed by blinded, independent observers, and formal interobserver agreement was not evaluated. This lack of blinded outcome assessment is acknowledged as a limitation.

Normality of continuous variables was formally assessed using the Shapiro-Wilk test before selecting parametric methods. Given the small group sizes (n = 6 and n = 10), the primary outcome comparison was additionally confirmed using the non-parametric Mann-Whitney U test (see Results). Reported cost variability reflects differences in the quantity of graft or template material dispensed per patient relative to a standard reference sheet size for each product, rather than variation in fixed catalogue unit prices; this basis for the reported standard deviations is clarified here in response to the reviewer’s comment.

## Results

### ADM manufacturing validation

**Figure 3:**
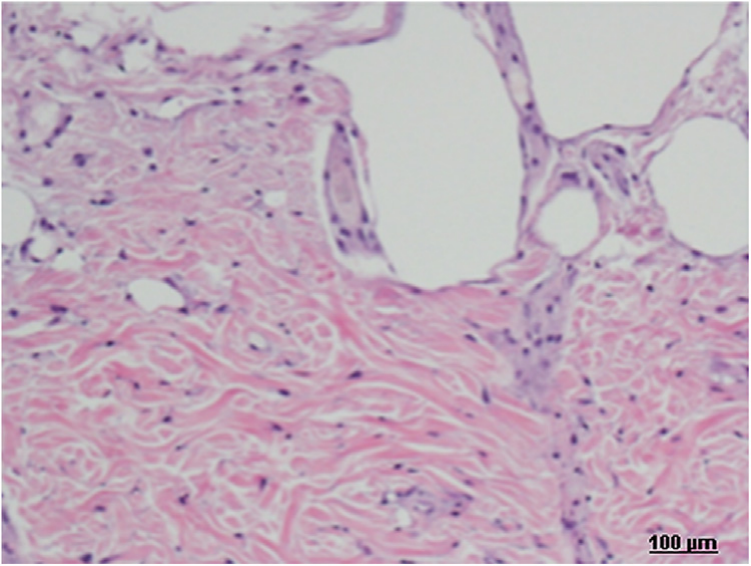
Optical photomicrograph of cryopreserved human dermis after decellularization, showing preservation of extracellular matrix architecture and absence of visible cellular components. H&E staining; scale bar = 100 µm.

Microbiological cultures obtained at all processing stages were negative for aerobic and anaerobic bacteria, fungi, and yeasts. Histological examination using haematoxylin and eosin staining confirmed the absence of cellular components and preservation of extracellular matrix architecture, validating the decellularization process (Figure 3).

### Patient characteristics

Patient ages ranged from 80 to 92 years, with male predominance in both groups. Mean age was 81.0 ± 1.3 years in the ADM group and 82.3 ± 3.6 years in the Integra® group, with no statistically significant between-group difference (p = 0.50, Mann-Whitney U test). Sex distribution did not differ significantly between groups (p = 0.60, Fisher's exact test). Histopathological diagnoses included basal cell carcinoma (BCC) in nine patients (ADM group: n = 4; Integra® group: n = 5) and squamous cell carcinoma (SCC) in seven patients (ADM group: n = 2; Integra® group: n = 5); tumour type distribution was comparable between groups (p = 0.63, Fisher's exact test). Mean defect size was 3.4 ± 0.7 cm (maximum diameter) in the ADM group and 2.9 ± 0.8 cm in the Integra® group, with no significant difference between groups (p = 0.23). Individual patient-level data, including age, sex, diagnosis, defect size, treatment received, healing time, and complications, are provided in Supplementary Table S1.

### Surgical outcomes

**Figure 4:**
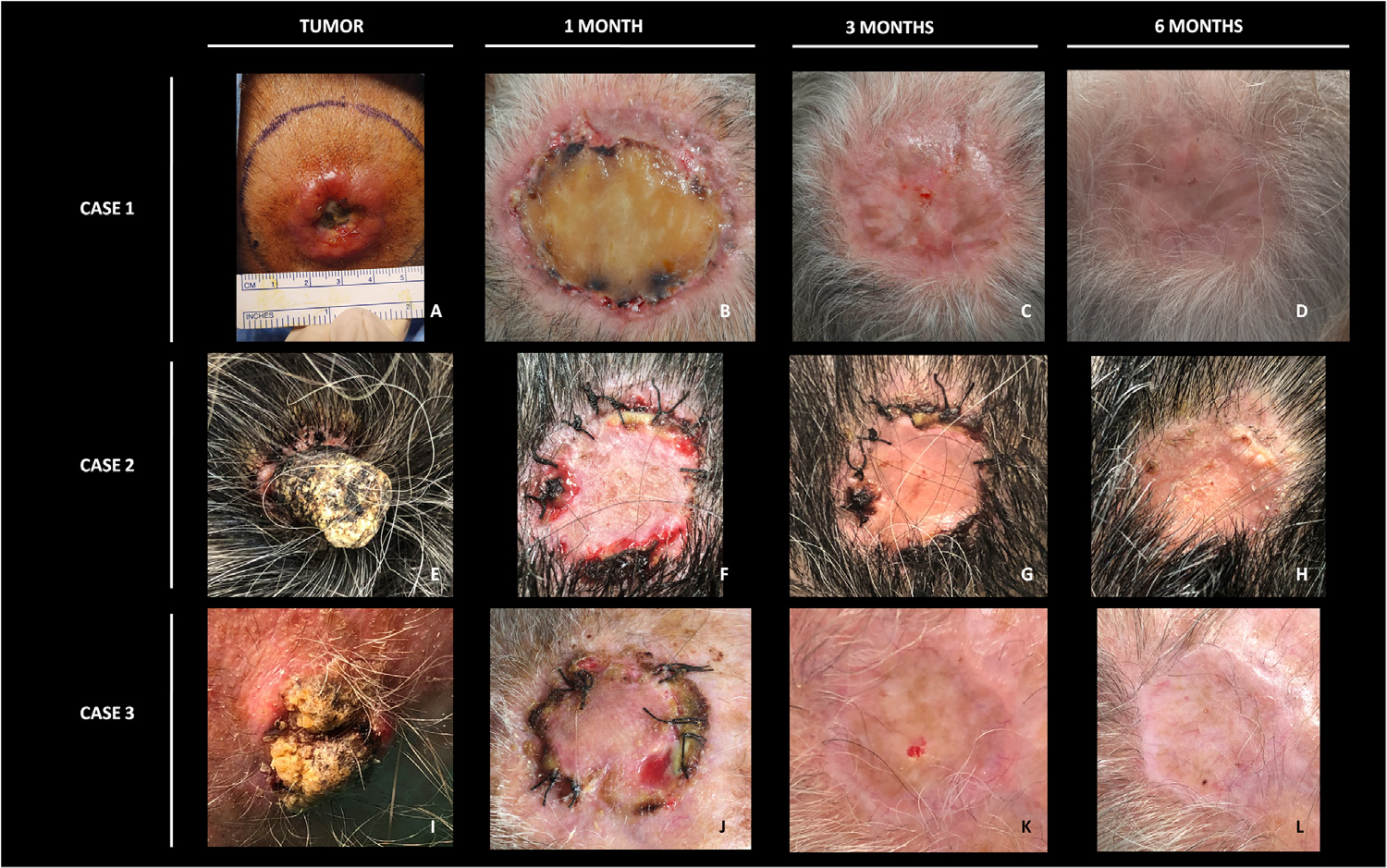
Clinical evolution. (A, E, I) Preoperative appearance. (B, F, J) One month postoperatively. (C, G, K) Three months postoperatively. (D, H, L) Six months postoperatively, demonstrating complete defect healing.

Index operative time ranged from 35 to 45 minutes and was comparable between groups (Table 1). All grafts integrated successfully; no graft failures occurred in either group. Representative clinical evolution at 1, 3, and 6 months is shown in Figure 4. One surgical site infection occurred in the ADM group, successfully managed with oral antibiotics; no infections occurred in the Integra® group (difference not statistically significant, p = 0.39).

**Table 1:** Clinical and operative outcomes by reconstructive material.

**Variable**	**ADM group (n=6) Mean ± SD**	**Integra® group (n=10) Mean ± SD**	**p value**
Age (years)	81.0 ± 1.3	82.3 ± 3.6	0.50
Defect size (cm)	3.4 ± 0.7	2.9 ± 0.8	0.23
Index operative time (min)	43.5 ± 3.4	39.5 ± 3.1	0.42
Healing time (months)	1.8 ± 0.3	3.5 ± 1.4	0.01*
Infection, n (%)	1 (16.7%)	0 (0%)	0.39
Graft failure, n (%)	0 (0%)	0 (0%)	—
Material cost (€)	500 ± 50	1759 ± 150	<0.001*

*Statistically significant (p < 0.05). SD: standard deviation; ADM: acellular dermal matrix. Defect size reported as maximum diameter in centimetres. Cost comparison based on a standard 10 × 12.5 cm sheet of each product; material costs exclude indirect expenditure (outpatient visits, dressing changes).

The primary endpoint, time to complete epithelialization, was significantly shorter in the ADM group (1.8 ± 0.3 months) than in the Integra® group (3.5 ± 1.4 months), with a mean difference of 1.7 months (95% CI 0.48–2.92; p = 0.01). Cohen's d was 1.54, indicating a large effect size. The post-hoc power analysis for the primary outcome estimated a power of 0.64; this value is reported only as contextual information and was not used to infer statistical validity. No significant difference was observed in defect size between groups (p = 0.23). Confirmatory non-parametric analysis using the Mann-Whitney U test supported the parametric comparison (U = 3.0, p = 0.004), indicating that the observed difference in healing time between groups was robust to the choice of statistical test.

### Cost analysis

The mean material cost of reconstruction using the tissue bank–derived ADM was approximately €4/cm² (€500 per 10 × 12.5 cm graft), compared with €14/cm² for Integra® (€1,759 per equivalent area; p < 0.001), representing a reduction of approximately 72% in direct material expenditure. As clarified in the methods, the reported cost variability corresponds to differences in the quantity of graft/template material dispensed relative to a standard 10 × 12.5 cm reference sheet for each defect, rather than variation in fixed catalogue unit prices.

## Discussion

This multicentre comparative pilot study suggests that single-stage scalp reconstruction using a tissue bank–manufactured human ADM may provide a feasible and lower-cost reconstructive strategy for elderly patients with oncologic scalp defects, with shorter observed time to epithelialization than Integra® in this small cohort. Given the small sample size and observational, non-randomised design, these results should be regarded as preliminary and hypothesis-generating rather than conclusive evidence of the superiority of either material.

Nonmelanoma skin cancer is among the most common malignancies affecting the scalp in elderly populations, and wide local excision often creates full-thickness defects involving the periosteum.^1^^,^^2^ In this context, the reconstructive surgeon must balance oncologic radicality with the physiological tolerance of patients who may be poor candidates for prolonged anesthesia or complex flap procedures. Single-stage ADM-based reconstruction offers a compelling solution: it avoids the staged protocols required by bilayer templates, reduces operative complexity, and limits donor site morbidity.

Acellular dermal matrices support wound healing through neovascularization, minimal inflammatory response, and resistance to infection.^9^^,^^10^ Their decellularization process removes immunogenic donor cellular components while preserving the extracellular matrix architecture, collagen scaffold, elastin, laminin, fibronectin, and glycosaminoglycans, structural proteins that contribute to cellular adhesion, proliferation, and tensile strength.^11^, ^12^, ^13^, ^14^, ^15^ In our protocol, detergent-based decellularization was intentionally avoided, preserving the intrinsic mechanical and biological properties of the human dermis without requiring gamma-irradiation.^9^

Integra® remains one of the most widely used commercial dermal substitutes for scalp reconstruction.^16^^,^^17^ Its bilaminate structure, a bovine collagen–glycosaminoglycan inner layer and an outer silicone membrane, typically requires a two-stage surgical procedure, with neodermis formation over 2–3 weeks before the silicone layer can be removed and a split-thickness skin graft applied.^18^^,^^19^ Infection rates of 13–15% have been reported with Integra® in scalp applications.^20^^,^^21^ In our series, no infections occurred in the Integra® group, which may reflect the relatively small sample size and careful patient selection.

The significantly shorter healing time in the ADM group (1.8 vs. 3.5 months) reflects two complementary factors. First, the inherent procedural advantage of single-stage reconstruction: while the ADM protocol allows immediate skin grafting at the index operation, the Integra® protocol mandates a second-stage procedure after neodermis formation (typically 2–3 weeks), which structurally extends the total treatment timeline regardless of the biological properties of either material. Second, the biological properties of human-derived matrices, including preserved growth factors, cytokines, and structural proteins, may confer additional healing advantages over cross-linked xenogeneic scaffolds. Mantelakis et al.^7^ demonstrated reliable single-stage graft integration and favourable aesthetic outcomes using a decellularised cadaveric dermis for nonmelanoma scalp and head and neck cancers. Campagnari et al.^22^ similarly reported comparable graft take and wound healing with human ADMs used instead of Integra® in oncologic reconstructive settings, consistent with the biological advantage of preserving native extracellular matrix components over cross-linked xenogeneic scaffolds.

The cost differential observed in this study has important implications for publicly funded healthcare systems. The tissue bank–derived ADM costs approximately €4/cm², compared with €14/cm² for Integra®, a 72% reduction in direct material expenditure. In health systems where high-cost biological implants require specific formulary approval, the availability of an equivalent product manufactured within the public tissue banking network can facilitate more equitable access to advanced reconstructive techniques. The development of this ADM by the Community Blood and Tissue Center of the Principality of Asturias exemplifies a circular economy model applied to biomedical tissue processing, transforming altruistic skin donations into clinically valuable scaffolds at a fraction of commercial costs.^7^^,^^8^

Several limitations of this study should be acknowledged. The sample size was small (n = 16), and this study is best interpreted as a multicentre pilot study providing proof-of-concept data and a basis for planning future confirmatory trials, rather than as a study with sufficient power for definitive conclusions. Post-hoc power for the primary outcome was 0.64; however, confidence intervals and effect size estimates are more informative for interpretation, and any future prospective randomised controlled trial should use an a priori sample size calculation. The observational, non-randomised design introduces potential selection bias; treatment allocation was determined by product availability during the early clinical implementation phase of the tissue bank–manufactured ADM, and the groups were comparable only for the baseline variables that were recorded. It should also be noted that part of the healing time difference between groups reflects a structural feature of the Integra® protocol itself, which mandates a two-stage procedure; the single-stage design of the ADM reconstruction therefore offers an inherent procedural advantage that must be considered when interpreting outcome differences. The study population was exclusively elderly patients with NMSC, which limits generalisability to younger patients or other anatomical locations. The cost analysis encompassed direct material costs only, excluding indirect healthcare expenditure such as additional outpatient visits associated with longer healing times in the Integra® group. Despite these limitations, this study presents several strengths: the introduction of a lower-cost, tissue bank–derived human ADM for head and neck oncologic reconstruction; the demonstration of reliable single-stage wound coverage with complete graft integration in this small cohort; the multicentre design that reduces single-institution bias; and the detailed characterisation of the manufacturing and decellularisation process.

An additional limitation concerns baseline comparability: beyond age, sex, tumour diagnosis and defect size, other variables potentially affecting wound healing, smoking status, diabetes, peripheral vascular disease, anticoagulant therapy, immunosuppression, previous radiotherapy, ASA score, and the Charlson Comorbidity Index, were not systematically recorded, and their absence limits formal assessment of comparability between the ADM and Integra® groups. Follow-up was limited to the healing period and a 6-month visit; longer-term outcomes such as contour stability, graft contraction, scar quality, patient-reported satisfaction, and late complications were not systematically assessed and represent an important focus for future prospective evaluation of this ADM. Histological characterisation of the processed ADM was limited to haematoxylin-eosin staining confirming decellularization; quantitative DNA content, collagen and elastin preservation, residual cellularity, and mechanical testing were not formally assessed in this study.

Regarding the regulatory status of the tissue bank–derived ADM, the product is processed and released as a human tissue graft, not as a manufactured medical device, under the framework governing tissue and cell establishments in Spain and the European Union applicable during the study period (Directive 2004/23/EC and its implementing directives). Its clinical use is authorised under the regional tissue bank's establishment licence. The new EU Regulation on substances of human origin (Regulation (EU) 2024/1938) will require alignment during its implementation period, and analogous production in other European tissue establishments would require local validation and specific regulatory authorisation in each jurisdiction.

Beyond the clinical outcomes, the manufacturing model described in this study represents a meaningful contribution to the emerging paradigm of circular economy in healthcare. Within this framework, altruistic skin donations—a biological resource generated through routine cadaveric tissue banking—are transformed into clinically validated acellular dermal matrices through a fully auditable, non-profit manufacturing pathway. This process creates measurable clinical value from a material that would otherwise not reach therapeutic use, embodying the core principles of the circular bioeconomy: resource efficiency, waste reduction, and value retention within closed biological cycles. The model eliminates commercial intermediaries, enables direct cost control within the public health system, and aligns biological implant production with broader sustainability goals in healthcare. Crucially, the regulatory infrastructure required for this approach is already in place across European tissue establishments with existing skin banking capacity; hospitals operating within such networks could adopt analogous decellularisation protocols to produce human ADMs locally, reducing reliance on commercial xenogeneic or allogeneic substitutes and improving equitable access to advanced reconstructive materials for patients who may not benefit from them under current commercial pricing structures.

## Conclusions

In this multicentre pilot study, reconstruction of oncologic scalp defects using a tissue bank–derived human acellular dermal matrix was associated with successful graft integration, shorter observed time to epithelialization, and lower direct material costs than Integra® in elderly patients with nonmelanoma skin cancer. These findings provide proof-of-concept evidence and may support the design of future randomised controlled trials. Integration of tissue bank–manufactured ADMs into reconstructive surgery protocols may expand access to advanced biological scaffolds in publicly funded healthcare systems, but the present findings should be considered preliminary and hypothesis-generating. Larger, prospective, and ideally randomised studies incorporating standardised comorbidity assessment and long-term outcome measures are required before broader clinical adoption can be recommended.

## Statements and additional information

### Author contributions (CRediT)

Conceptualization and supervision: LG, LJ. ADM manufacturing: MP, AM, FS. Preoperative planning: LG, LR, CS, LJ. Surgery: LG, LJ, LR, CS. Postoperative follow-up: LG, DR. Writing and editing: LG, MP, LJ. Critical revision and final approval: all authors.

### Ethical approval

The study was conducted in accordance with the principles of the Declaration of Helsinki and approved by a regional ethics committee (approval code: 2021.547).

### Informed consent

Written informed consent for treatment and use of clinical photographs for publication was obtained from all patients.

### Funding

This research received no specific grant from any funding agency in the public, commercial, or not-for-profit sectors. The acellular dermal matrix was developed and manufactured within the Community Blood and Tissue Center of the Principality of Asturias, integrated within the Spanish public health system.

## Declaration of competing interest

The authors declare no conflicts of interest.

## Data Availability

The data that support the findings of this study are available from the corresponding author on reasonable request, subject to institutional ethics committee conditions.

## Appendix Supplementary materials

Image, application 1
