# Supplementary material for: Human acellular dermal matrix versus Integra® for single-stage oncologic scalp reconstruction: A multicentre cohort study
Source: JPRAS Open. 2026 Jul 23;51:520–30. doi: 10.1016/j.jpra.2026.07.026 (PMC13470571; doi:10.1016/j.jpra.2026.07.026)
Supplement: Supplementary file 1 [file mmc1.pdf]

Patient ID	Group	Age (years)	Sex	Diagnosis	Defect size (cm, max. diameter)	Treatment	Healing time (months)	Complications
P01	ADM	80	M	BCC	2.5	ADM + STSG	1.5	None
P02	ADM	81	F	BCC	3.0	ADM + STSG	1.8	None
P03	ADM	80	M	SCC	4.0	ADM + STSG	2.3	Surgical site infection (oral antibiotics)
P04	ADM	82	M	BCC	3.2	ADM + STSG	1.6	None
P05	ADM	80	F	SCC	4.5	ADM + STSG	2.0	None
P06	ADM	83	M	BCC	3.2	ADM + STSG	1.6	None
P07	Integra®	81	M	BCC	2.0	Integra® + STSG (2 stages)	2.0	None
P08	Integra®	80	M	BCC	2.5	Integra® + STSG (2 stages)	2.5	None
P09	Integra®	82	M	BCC	3.0	Integra® + STSG (2 stages)	3.0	None
P10	Integra®	80	F	BCC	2.2	Integra® + STSG (2 stages)	3.5	None
P11	Integra®	83	M	BCC	3.5	Integra® + STSG (2 stages)	4.0	None
P12	Integra®	80	M	SCC	3.8	Integra® + STSG (2 stages)	4.5	None
P13	Integra®	81	F	SCC	2.9	Integra® + STSG (2 stages)	2.5	None
P14	Integra®	92	M	SCC	4.5	Integra® + STSG (2 stages)	6.0	None
P15	Integra®	83	M	SCC	2.0	Integra® + STSG (2 stages)	2.0	None
P16	Integra®	81	M	SCC	2.5	Integra® + STSG (2 stages)	5.0	None

Supplementary Table S1. Individual patient-level data

STSG: split-thickness skin graft. BCC: basal cell carcinoma; SCC: squamous cell carcinoma. Defect size reported as maximum diameter in centimetres. Healing time: time to complete epithelialization from the index reconstructive operation. "None" indicates no complication recorded; no graft failures occurred in either group.
